# Loss Aversion and Learning in Professional Golf Putting

**DOI:** 10.3390/bs16030321

**Published:** 2026-02-26

**Authors:** Dongyoup Lee

**Affiliations:** College of Business Administration, Kookmin University, Seoul 02707, Republic of Korea; dlee@kookmin.ac.kr

**Keywords:** golf putting, behavioral bias, loss aversion, self-regulation, adaptive learning

## Abstract

This paper provides new field-based evidence on loss aversion and short-run learning using high-frequency performance data from professional golf. Leveraging over 100,000 putts recorded during the 2020 Korea Professional Golfers’ Association (KPGA) Tour, I examine how professional golfers adjust their putting behavior in response to reference-dependent incentives and immediate feedback. The structure of golf creates a natural empirical setting to test behavioral predictions: scoring rules establish salient reference points (e.g., par), while putting decisions are discrete, individually executed, and financially consequential. I find that players are significantly more likely to convert par-saving putts than birdie attempts from equivalent distances, consistent with loss aversion and reference-dependent preferences. Par putts are also executed more aggressively, but players regulate pace to avoid costly three-putt errors, indicating strategic self-regulation under loss-framed incentives. In addition, I document robust evidence of within-hole learning: second putts—taken shortly after the first under near-identical conditions—exhibit substantially higher success rates. These patterns are confirmed in logistic regression models with nonlinear distance controls and player fixed effects. This performance gap persists across scoring frames and aligns with models of reinforcement learning and dynamic belief updating. The findings illustrate how behavioral biases and adaptive learning interact in high-stakes, real-world decisions and highlight the value of professional sports data for testing core theories in behavioral economics.

## 1. Introduction

*“Drive for show, putt for dough.”*

*—Bobby Locke*

Understanding how individuals make decisions under uncertainty is central to economics. While classical models assume stable preferences and rational optimization, a growing body of research in behavioral economics demonstrates that real-world decision-making is systematically influenced by psychological biases, cognitive limitations, and learning from experience. Among these, loss aversion—the tendency to weigh losses more heavily than equivalent gains (Kahneman & Tversky, 1979)—and adaptive learning—the process of updating beliefs and strategies based on feedback (Erev & Roth, 1998; Camerer & Hua Ho, 1999)—have been shown to affect behavior in domains ranging from financial markets to consumer choice. Yet despite their theoretical importance, empirical identification of these behavioral patterns remains challenging, particularly in naturally occurring environments where decisions are repeated, incentives are salient, and outcomes are observable. This paper contributes to this literature by providing new empirical evidence on loss aversion and short-run learning using novel high-frequency decision data.

Professional golf offers a uniquely valuable setting for studying behavioral decision-making in the field. Golfers repeatedly face discrete, high-stakes choices—such as whether and how to attempt a putt—where outcomes are binary (make or miss), incentives are clearly defined, and performance is individually observable. Putting decisions, in particular, are made independently of other players and involve immediate feedback, minimizing concerns about strategic interaction or external attribution. Importantly, each green presents unique environmental variation (e.g., slope, speed, grain), requiring players to adapt their expectations and strategies with limited prior information. This structure creates a natural opportunity to test models of experience-based learning as players refine their judgment over the course of play. Furthermore, the scoring system in golf establishes psychologically salient reference points—most notably par—that facilitate empirical tests of reference-dependent preferences and loss aversion in high-stakes environments (Pope & Schweitzer, 2011). Leveraging detailed shot-level data from the Korea Professional Golfers’ Association (KPGA) Tour, this paper examines how professional golfers adjust their putting strategies based on scoring context and feedback, revealing evidence of both behavioral bias and dynamic learning—even among elite performers.

Among the various components of professional golf performance, putting plays a disproportionately influential role in determining scoring outcomes. Although driving, iron play, and wedge shots contribute to a player’s position and opportunities, it is putting that directly translates shot execution into realized score. On average, putts account for over one-third of total strokes in a professional round, and their relative scarcity in total shot distance is offset by their marginal impact on score per stroke. Broadie (2018) shows that although tee-to-green performance explains a larger share of average scoring differentials, variation in tournament outcomes is disproportionately influenced by fluctuations in putting performance due to its relatively high volatility. Unlike driving or iron play, putting outcomes are not easily recoverable: a missed short putt immediately results in a higher score, whereas a missed fairway or green in regulation can often be mitigated through subsequent strokes. Consequently, the marginal cost of error is highest on the green. 

Motivated by these considerations, this paper addresses two questions. First, do professional golfers exhibit reference-dependent behavior consistent with loss aversion in putting decisions? Second, do players exhibit short-run, within-hole learning by incorporating immediate feedback from prior putts?

To examine these questions, I construct a novel dataset using detailed shot-level performance records from the 2020 season of the KPGA Tour. The dataset includes over 100,000 putts across nine professional tournaments and contains rich information on each shot, including distance to the hole, success or failure, remaining distance when missed, scoring context (birdie, par, or bogey), and the order of the putt within the hole. This structure allows for two complementary empirical strategies. First, I test for loss aversion by comparing putt conversion rates and execution strategies across scoring frames, controlling for putt distance. Second, I investigate short-run learning by comparing the success rates of first and second putts at matched distances, exploiting the within-player, within-hole variation to control for environmental and individual-level heterogeneity. Formal regression models incorporating nonlinear distance effects and player fixed effects provide statistical validation of the descriptive patterns. This approach provides a clean identification of both reference-dependent behavior and experience-based learning in a natural, performance-driven environment. 

This paper makes two main contributions. First, I provide empirical evidence of loss-averse behavior in professional putting. Conditional on distance, players are significantly more likely to convert par-saving putts than birdie attempts. Regression analysis shows that par putts decline less steeply in success probability as distance increases, implying that the par advantage widens with difficulty. Moreover, par putts are executed more aggressively than birdie putts, yet players simultaneously regulate their pace to avoid follow-up errors, resulting in no measurable difference in three-putt likelihood across scoring frames. These patterns are consistent with strategic self-regulation in the presence of loss aversion and anticipated regret. Second, I document robust evidence of short-run, within-hole learning. Using within-player variation, I show that second putts—taken immediately after the first, under identical environmental conditions—are converted at significantly higher rates than first putts of the same distance. This second-putt advantage remains economically large after controlling for player heterogeneity. The results align with models of reinforcement learning and belief updating, suggesting that even elite professionals adapt rapidly in response to immediate feedback. Together, these findings offer field-based evidence of both behavioral bias and adaptive learning in a high-skill, high-incentive environment, and demonstrate how professional sports data can be used to test and refine core theories in behavioral economics. 

The remainder of the paper is organized as follows. Section 2 reviews the related literature. Section 3 describes the data and presents summary statistics. Section 4 reports the empirical results on loss aversion and examines evidence of short-run learning. Section 5 concludes. 

## 2. Literature Review

This paper contributes to two primary strands of literature in behavioral economics: reference-dependent preferences and loss aversion, and learning from experience under uncertainty. A growing body of empirical research has documented how individuals’ decisions deviate from classical rational choice models when outcomes are framed relative to salient reference points. Prospect theory (Kahneman & Tversky, 1979) formalized the notion that individuals are loss averse—that is, they experience losses more acutely than equivalent gains. This insight has been widely applied in domains such as consumption (Abeler & Marklein, 2010), labor supply (Crawford & Meng, 2011), and performance under pressure (Gill & Prowse, 2012; Allen et al., 2017). Recent evidence further reinforces the empirical relevance of reference-dependent preferences across economic domains. Andersen et al. (2019) demonstrate that individual loss experiences have persistent effects on subsequent risk-taking behavior. Van Bilsen et al. (2020) develop a dynamic framework in which reference levels evolve endogenously, highlighting the intertemporal implications of loss aversion for consumption and portfolio choice. Complementing these findings, Brown et al. (2024) provide a meta-analysis of empirical estimates of loss aversion, documenting both its robustness and substantial heterogeneity across settings.

This paper also relates to a complementary literature on learning and belief updating in dynamic decision environments. In models of reinforcement learning (Erev & Roth, 1998) and experience-weighted attraction learning (Camerer & Hua Ho, 1999), agents adjust their strategies based on feedback from previous actions. Sutton and Barto (1998) similarly model learning as a process of updating expectations through real-time experience. These theories have been tested in various applied contexts, including education (Nguyen, 2019), online behavior (Goldfarb & Tucker, 2011), and consumer experimentation (Yao et al., 2012). More recent work highlights that learning may be shaped by cognitive constraints and motivational forces. Ambuehl et al. (2022) show that belief updating depends on deliberative competence and the structure of the decision environment, while Zimmermann (2020) demonstrates that motivated reasoning can systematically influence how feedback is interpreted. In sports, these dynamics are particularly salient: players operate in high-frequency decision environments with immediate performance feedback and meaningful incentives. While many learning studies rely on experimental data, field settings—especially those involving individual, repeated decisions—offer a promising avenue for empirical validation of adaptive behavior. 

In the context of sports—and golf in particular—Pope and Schweitzer (2011) provide direct evidence of loss aversion by analyzing PGA Tour data. They show that professional golfers are significantly more likely to make par-saving putts than equidistant birdie putts, consistent with par serving as a salient reference point. Recent evidence further supports reference-dependent behavior in professional golf. Elmore and Urbaczewski (2021) exploit U.S. Open holes reclassified from par 5 to par 4 without substantive redesign, isolating changes in the reference point while holding the task largely constant. They find that performance responds to the par designation, consistent with loss aversion and prospect theory. This evidence reinforces the role of par as a salient psychological benchmark and motivates additional tests in alternative tours and shot-level contexts. Loughran and Schultz (2004) explore the role of green elevation and visual perception in missed putts, while Peláez-Zuberbühler et al. (2021) examine biomechanical adjustments under varying performance pressure. These studies highlight the role of physical, perceptual, and psychological factors in shaping putting outcomes. Additionally, golf offers a rich structure for examining learning: players make sequential decisions with minimal strategic interdependence and receive immediate feedback on outcomes. McFall et al. (2009) and Guryan et al. (2009) use golf tournament data to study how performance and effort evolve in response to prior shots and social comparisons. This paper builds on these insights by using shot-level data to identify both reference-dependent behavior and within-hole learning, offering new evidence on how elite decision-makers adapt under real-time feedback and performance pressure. 

A related literature studies whether performance exhibits “streakiness” in professional golf, often distinguishing between hot-hand and cold-hand effects. Elmore and Urbaczewski (2018) examine hole-by-hole outcomes and report limited evidence of a hot hand but more consistent evidence of a cold hand. Stone and Arkes (2016) argue that reference points can generate serial correlation patterns that interact with momentum-like effects; in their framework, prospect-theoretic responses around salient benchmarks may dominate or mask hot-hand dynamics, particularly when outcomes are evaluated relative to par. More recently, Ehrlich et al. (2025) reassess momentum using fixed effects methods and recent PGA Tour seasons, offering updated evidence on the magnitude of hot- and cold-hand effects. Together, these studies suggest that asymmetric responses to negative outcomes—consistent with loss sensitivity—may be an important component of performance dynamics in golf, and they provide a useful complement to putt-level tests of reference dependence.

Beyond golf, a growing number of studies have used data from other sports to test behavioral economic theories in high-stakes, real-world environments. In soccer, Palacios-Huerta (2012) and Chiappori et al. (2002) use penalty kick data to examine equilibrium strategies and strategic behavior under pressure, while Bar-Eli et al. (2009) document deviations from optimal decision-making by goalkeepers, consistent with action bias. Camerer and Johnson (1991) and Miller and Sanjurjo (2018) investigate beliefs in the “hot hand” phenomenon, showing how players and observers may misinterpret random variation as momentum, reflecting biases in learning and expectation formation. Across these sports, researchers have leveraged structured data environments with frequent decision-making and well-defined incentives to test for reference dependence, loss aversion, learning, and bounded rationality. This paper contributes to that literature by focusing on golf, a domain that isolates individual decision-making and enables unusually clean identification of both psychological framing effects and feedback-driven learning. 

## 3. Data

To conduct the empirical analysis, this study utilizes detailed performance data from the 2020 season of the Korea Professional Golfers’ Association (KPGA) Tour. The data were obtained from CNPS, the official record-keeping agency of the KPGA. Table 1 summarizes the dataset, which includes nine of the eleven tournaments held in 2020 for which putting information is available. In total, the dataset comprises 35 rounds and contains hole-by-hole performance records for all participating players. Importantly, for each putt, the data contain granular information such as the distance to the hole, whether the putt was successful, and the remaining distance when the putt was missed—enabling precise analysis of putting behavior. On average, each tournament featured approximately 136 players. The average number of putts per hole ranged from 1.65 to 1.74, with a maximum of five putts recorded on two occasions. Across all tournaments, the dataset includes 104,281 individual putts. 

**Figure 1:**
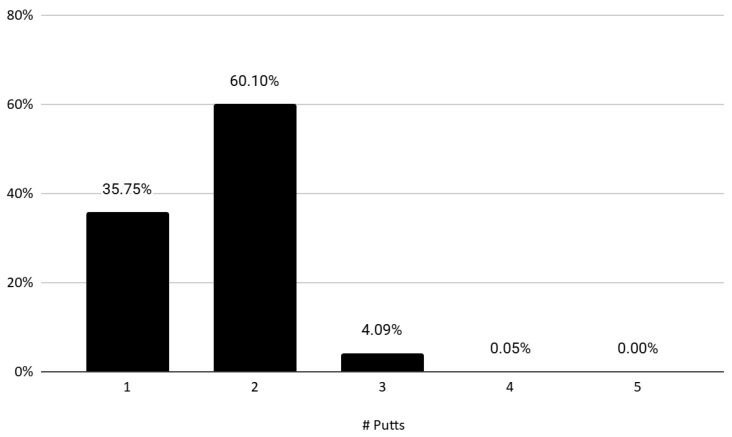
Distribution of the number of putts per hole.

Figure 1 presents the distribution of the number of putts per hole. Among a total of 61,903 hole-level observations, 35.75% were completed with a single putt. The majority of plays, 60.10%, resulted in two putts, while only 4.09% required three putts. Four- and five-putt occurrences are extremely rare. The average number of putts per hole across the sample is 1.68, indicating that most players are able to complete the hole with relatively few strokes on the green. In this context, recording a three-putt represents a significant deviation from the norm and may be critically detrimental to a player’s overall performance.

Table 2 presents the composition of the putt sample. Of the 104,281 total putts in the dataset, 41,863 (approximately 40%) were first-putt attempts for birdie or eagle. The second most frequent category consists of second-putt attempts for par, accounting for roughly 30% of the sample. These par-saving second putts typically follow missed birdie attempts. The observed make rate for first putts attempted for birdie or eagle is 24.43%, indicating that the majority of these opportunities result in a follow-up attempt to save par rather than a scoring conversion. There are 117 instances in the dataset where a hole begins with a birdie attempt but results in a three-putt—an outcome that players are particularly motivated to avoid.

**Figure 2:**
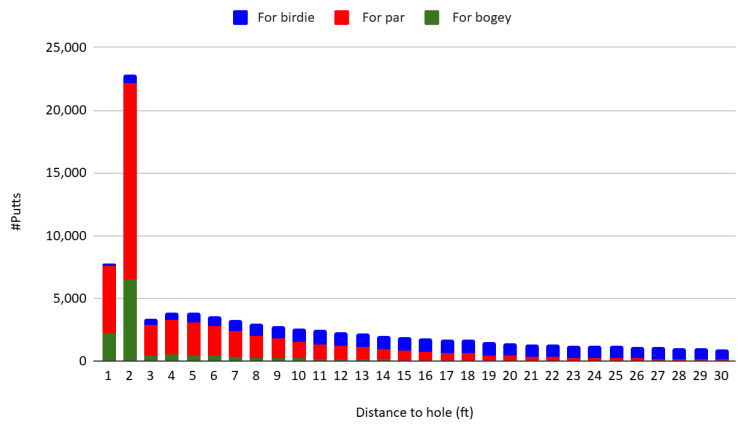
Distribution of putt distance to the hole for birdie, par, and bogey.

Figure 2 displays the distribution of putt distances to the hole, disaggregated by scoring context—specifically, whether the putt is for birdie or eagle, par, or bogey and worse. The majority of par and bogey putts are attempted from within two feet, comprising 43.44% and 65.67% of their respective categories. In contrast, only 2.21% of birdie putts are attempted from within two feet, indicating that birdie opportunities generally occur from substantially longer distances. This disparity reflects the sequential nature of play: many par putts attempted from close range follow missed birdie attempts from farther away. Overall, 29.41% of all putts in the sample are taken within two feet of the hole, the majority of which are par-saving attempts.

As shown in Figure 2, 72.96 percent of all putts in the sample are attempted from within 20 feet of the hole. The empirical analysis therefore focuses on putts shorter than 20 feet. In professional golf terminology, lag putting typically refers to substantially longer attempts—often beyond 20 to 25 feet—where the primary objective is distance control rather than holing out. By restricting attention to putts within 20 feet, the analysis largely excludes traditional lag-putting situations and instead concentrates on attempts that remain genuine scoring opportunities. 

## 4. Results

### 4.1. Loss Aversion and Strategic Self-Regulation

*“Golf is a game of misses. He who misses best wins.”*

*—Ben Hogan*

Professional golf provides an ideal empirical setting for testing the behavioral predictions of loss aversion. The structure of the game creates clearly defined reference points—most notably, par—which allow for clean comparisons of decision-making under gain-versus-loss framing. For example, a putt for birdie and a putt for par from the same distance are mechanically equivalent in difficulty, but differ in psychological framing: the birdie putt represents a potential gain, whereas the par putt is framed as the avoidance of a loss. According to prospect theory (Kahneman & Tversky, 1979), individuals are more sensitive to losses relative to a reference point than to equivalent gains. This paper directly tests the prediction using detailed shot-level data in KPGA.

**Figure 3:**
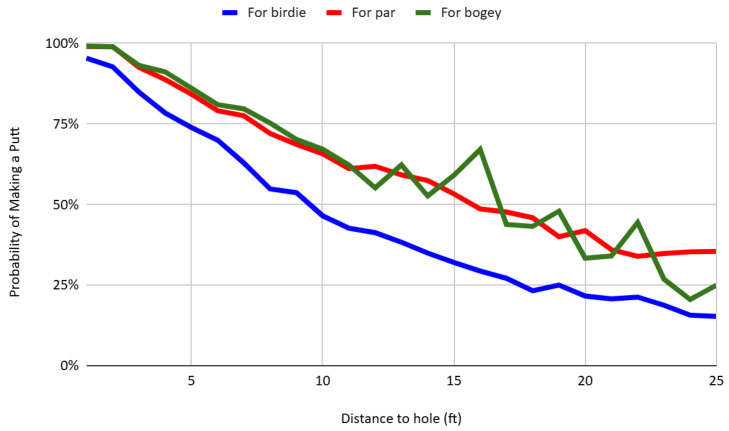
Putt success rates by distance and scoring context.

Figure 3 presents the probability of making a putt as a function of distance, separately for putts attempted for birdie, par, and bogey. The make percentage for birdie putts is consistently and significantly lower than for par- and bogey-saving putts at equivalent distances. For instance, at a distance of 10 feet, the conversion rate for par putts is 46.48 percent, which is 19.27 percentage points higher than the rate for birdie putts, and this difference becomes larger as the distance from the hole increases. This pattern is consistent with the findings of Pope and Schweitzer (2011), who document similar asymmetries in PGA Tour data. The results suggest that players are more likely to convert par-saving putts than birdie opportunities, conditional on distance. Such behavior is consistent with reference-dependent preferences and the predictions of loss aversion: when par is treated as the psychological reference point, missing a par putt is experienced as a loss, while failing to convert a birdie is framed as a foregone gain. As a result, players appear to allocate greater cognitive or emotional effort to avoiding losses than to achieving gains, resulting in observable performance differences across scoring contexts. These findings support a growing body of literature on reference dependence in goal pursuit and performance-based tasks (Gill & Prowse, 2012; Allen et al., 2017).

The disparity in conversion rates between birdie and par putts may be partially explained by differences in putting aggressiveness, defined as the player’s willingness to apply sufficient pace to reach or pass the hole. In standard models of optimal play, aggressiveness should be invariant to scoring context, conditional on distance. However, behavioral considerations suggest otherwise: under reference-dependent preferences, players may be more inclined to adopt aggressive strategies when attempting to avoid a perceived loss (e.g., missing a par putt) than when pursuing a gain (e.g., converting a birdie putt). Specifically, if players experience greater psychological disutility from missing a par putt than from missing a birdie putt, they may be more willing to risk overshooting the hole in the former case.

**Figure 4:**
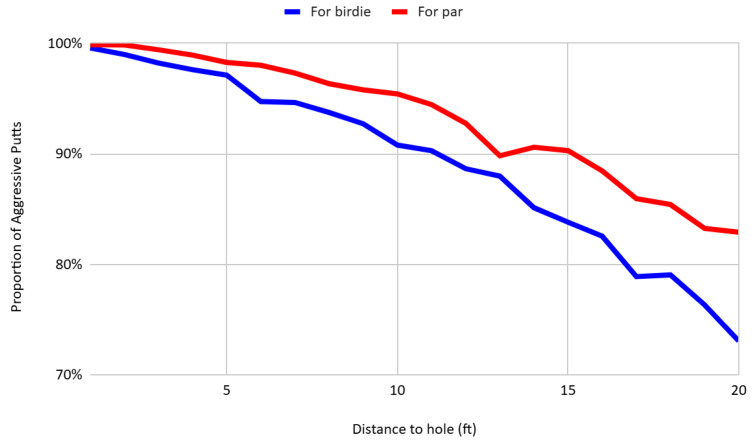
Aggressiveness on the greens for birdie and par by distance.

Figure 4 illustrates aggressiveness on the greens by distance for birdie and par. It reveals that aggressiveness is systematically higher for par putts than for birdie putts at equivalent distances. At 10 feet from the hole, the aggressiveness rate for par putts exceeds that of birdie putts by 4.62 percentage points. Moreover, this difference increases with distance, suggesting that players disproportionately moderate their pace on longer birdie attempts relative to par-saving situations. This pattern is consistent with the findings of Pope and Schweitzer (2011) and aligns with the behavioral prediction that players exert greater effort when attempting to avert a loss relative to when pursuing a potential gain.

These findings also imply that execution strategy, and not merely mechanical skill or green conditions, contributes to observed asymmetries in putting outcomes. The variation in aggressiveness across scoring frames provides further evidence of loss-averse behavior in professional golfers, even in settings with high levels of skill and experience.

**Figure 5:**
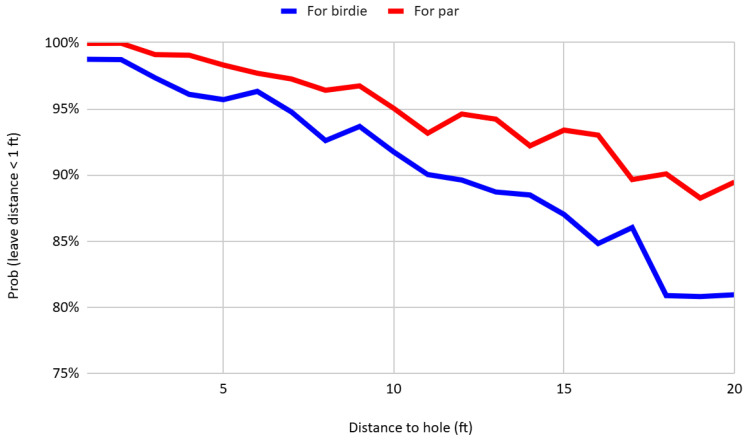
Probability that missed putts for birdie and par finish within one foot of the hole by distance.

Nonetheless, further analysis reveals a more complex psychological process. Figure 5 presents the probability of missed putts finishing within one foot of the hole for birdie and par by distance. While par putts are executed more aggressively than birdie putts as evidenced by longer leave distances on average, the results reveal that missed par putts are more likely to finish within one foot of the hole than missed birdie putts. At first glance, this result appears inconsistent with the loss-aversion framework, which would predict greater risk-taking (and thus longer misses) on par putts. One possible explanation is that players facing a par putt internalize not only the loss associated with missing the current shot but also the anticipated disutility of a three-putt, which could result in a double bogey or worse. In this interpretation, players adjust their pace to avoid leaving themselves a difficult follow-up putt, thus moderating their execution despite a generally more aggressive strategy. This behavior is also consistent with regret aversion, whereby players anticipate the emotional cost of making a compounding error and consequently exercise greater control over pace, particularly under pressure.

**Figure 6:**
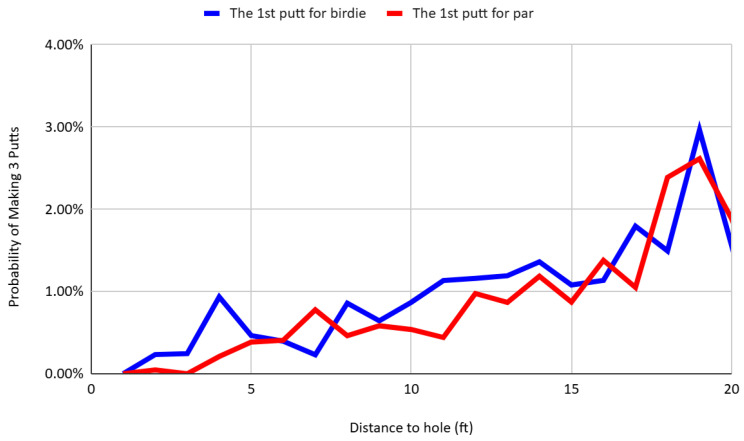
Probability of making three putts after the first attempt for birdie or par by distance.

In line with this reasoning, the probability of making three putts does not differ meaningfully between par and birdie contexts, as shown in Figure 6. This provides further evidence of strategic self-regulation. Although players display different levels of aggressiveness and initial success rates depending on the scoring frame, they appear to manage their overall risk of multi-putting such that cumulative error rates remain stable. This behavior is consistent with models of bounded rationality (Gabaix, 2014) and satisficing (Caplin et al., 2011), in which individuals operate under cognitive constraints but adopt heuristics that prevent performance breakdowns. It may also reflect forward-looking optimization, where players internalize the expected value of future outcomes and adjust their strategies to minimize downside risk, even when operating under loss-framed incentives. In this sense, they “miss best,” as Ben Hogan suggested—not by eliminating error entirely, but by optimizing how error occurs. That is, players shape the distribution of their misses to avoid large penalties (e.g., three-putts), even if that means adjusting their execution strategies dynamically across scoring frames.

Taken together, the empirical evidence presented here is consistent with the central tenets of prospect theory and other behavioral models incorporating loss aversion and strategic self-regulation (Barberis, 2013; Rabin, 2000). Players appear to exert greater effort and adopt more aggressive strategies when avoiding a loss relative to achieving a gain, but also internalize the risk of future errors and adjust their play accordingly. The result is a behavioral equilibrium in which execution varies by context, but overall performance risk—particularly the probability of three-putting—remains tightly managed. This nuanced behavioral profile underscores the value of using professional sports data to test and refine theories of decision-making under uncertainty, particularly when individual performance is observable at high frequency and under meaningful financial incentives.

### 4.2. Within-Hole Learning and Behavioral Updating

*“Putting is a game within a game.”*

*—Bobby Locke*

Golf provides a particularly advantageous setting for empirically studying learning behavior in repeated, high-stakes decision environments. Unlike many real-world domains, where learning is difficult to identify due to latent heterogeneity, sparse feedback, or overlapping strategic interactions, professional golf offers a highly structured sequence of discrete, individually executed tasks. In particular, putting decisions are well-defined, outcome-based, and incentive-compatible: each putt constitutes a standalone choice with binary outcomes (make or miss), and performance translates directly into scoring and financial rewards. These features ensure that any observed behavioral adjustments reflect genuine, performance-driven learning rather than noise or extrinsic incentives.

Putting is especially well-suited for examining short-run learning dynamics. Although the physical execution of a putt remains stable, the informational environment varies considerably across holes due to differences in green slope, speed, grain, and firmness. Players must extract this information in real time and adapt their execution accordingly. Critically, this learning is endogenous to the game environment: the outcome of a first putt provides direct, personalized feedback about how the green behaves—how much the ball breaks, how fast it rolls, and how pace translates to distance.

This structure creates a natural setting to test whether learning occurs within the span of a single hole. Because the first and second putts are taken in close temporal succession, on the same surface, and under identical environmental conditions, it is possible to hold fixed most sources of contextual heterogeneity. Moreover, by comparing conversion rates across putts of equivalent distance, one can control for the most important mechanical determinant of difficulty, thereby isolating the impact of learning from variation in putt length.

**Figure 7:**
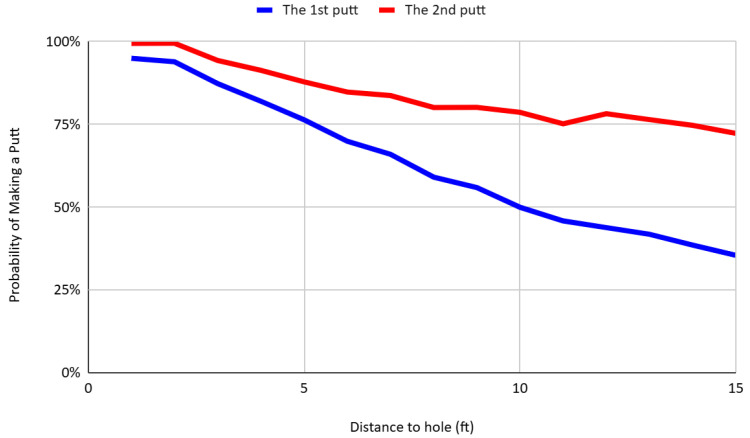
Putt success rates of first and second attempts by distance.

To empirically identify the effect of learning, I compare the probability of holing the first and second putts, conditional on putt distance. Figure 7 presents the conversion rates for first and second putts across matched distance intervals. The results reveal a clear and statistically significant pattern: the success rate for second putts consistently exceeds that of first putts at all distances. Moreover, the magnitude of this difference increases with distance from the hole, suggesting that the informational value of the first putt grows as the complexity of the green reading task increases. For example, at a distance of 10 feet, the conversion rate for second putts is 78.67%, compared to just 49.97% for first putts—a gap of nearly 29 percentage points. This pattern implies within-hole learning, whereby players incorporate the outcome of the first putt to improve their execution on the second attempt.

This finding is consistent with models of short-term, adaptive learning under uncertainty, such as reinforcement learning (Erev & Roth, 1998) and experience-weighted attraction models (Camerer & Hua Ho, 1999), in which agents respond to recent outcomes by updating beliefs and adjusting behavior. Because both putts are taken under near-identical conditions, the improvement in performance cannot be attributed to selection or unobserved heterogeneity. Rather, the result reflects real-time behavioral adjustment based on feedback. In this sense, putting provides a natural experiment for observing adaptive learning at the level of individual decisions. As in Erev and Roth’s (1998) framework, players appear to reinforce successful actions and adjust away from errors based on the most recent payoff-relevant experience. Similarly, the observed within-hole learning mirrors the Bayesian updating of attraction weights in Camerer and Hua Ho’s (1999) model, in which agents weigh past outcomes according to recency and salience.

**Figure 8:**
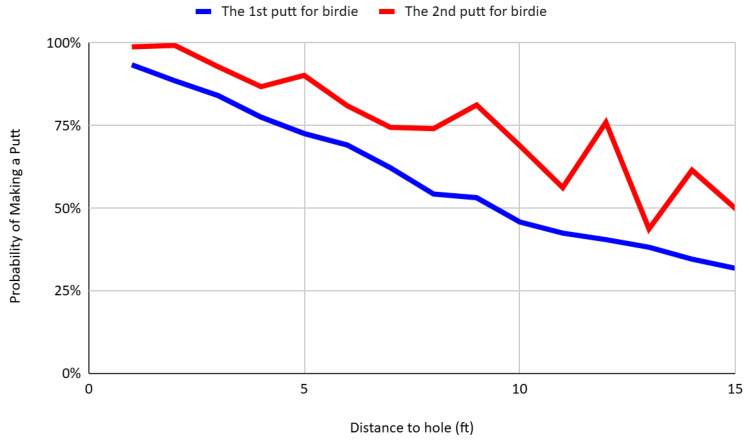
Putt success rates of first and second birdie attempts by distance.

**Figure 9:**
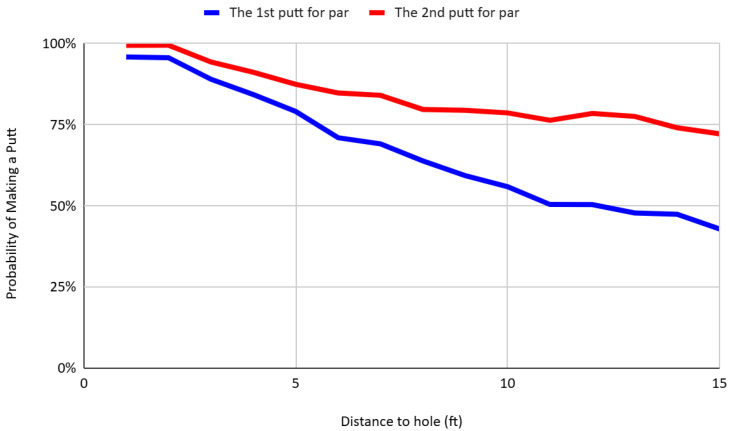
Putt success rates of first and second par attempts by distance.

**Figure 10:**
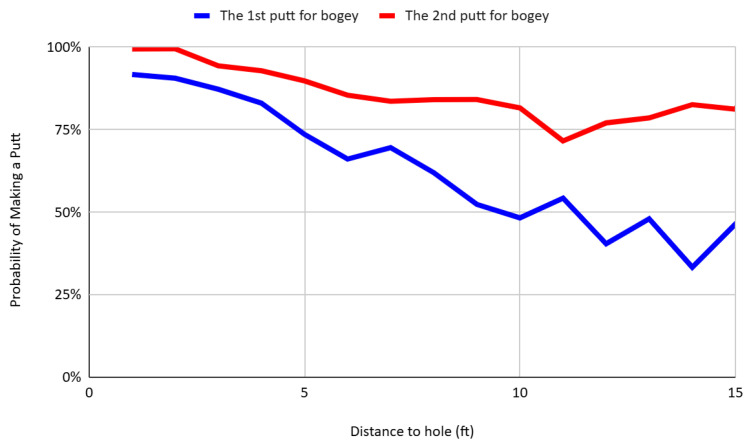
Putt success rates of first and second bogey attempts by distance.

To assess the robustness of the main finding, I conduct a disaggregated analysis of first and second putt performance across different scoring contexts—specifically, putts for birdie, par, and bogey—conditional on distance. Figure 8, Figure 9 and Figure 10 present the conversion rates by distance for each scoring category. In all cases, the pattern persists: second putts exhibit systematically higher success rates than first putts at comparable distances. For instance, at a distance of 10 feet, the conversion rates for second putts aimed at birdie, par, and bogey are 68.96%, 78.66%, and 81.63%, respectively—substantially higher than the corresponding first putt success rates of 45.85%, 55.91%, and 48.28%. This result holds across all scoring frames, despite differences in psychological incentives, strategic considerations, and shot framing. The consistency of this pattern across contexts reinforces the interpretation that players engage in rapid, within-hole learning by incorporating feedback from the outcome of the first putt to improve the execution of the second.

This approach offers a credible and transparent identification strategy for short-run learning in naturalistic settings. By exploiting within-player, within-hole variation, the analysis avoids common confounds associated with cross-sectional comparisons or tournament-level learning trends. More broadly, the results contribute to the growing literature on learning under uncertainty, showing that even highly trained professionals adapt their behavior dynamically in response to immediate feedback—a behavioral pattern relevant not only for sports but also for broader economic environments characterized by high-frequency decision-making and context-specific uncertainty.

### 4.3. Regression Evidence

To formally assess the statistical significance and magnitude of the patterns documented in Section 4.1 and Section 4.2, I estimate logistic regression models of putt success. The regression analysis uses 90,882 observations, restricted to birdie and par attempts and to first and second putts. This restriction facilitates direct comparison across scoring frames while isolating within-hole learning. The dependent variable, Success(i,p), is an indicator equal to one if the putt is holed and zero otherwise. The baseline specification is:

logit(Pr[Success(i,p)]) = α + βln[Distance(i,p)] + θPar(i,p) + φSecond(i,p) + δln[Distance(i,p)] × Par(i,p) + ψln[Distance(i,p)] × Second(i,p) + μ(p),(1)

where Distance(i,p) denotes the distance to the hole (in feet) for putt i by player p. Par(i,p) is an indicator equal to one for par-saving putts (with birdie putts as the omitted category); Second(i,p) equals one for second putts; and p represents player fixed effects that control for persistent heterogeneity in putting ability. Standard errors are clustered at the player level.

Table 3 reports the estimation results using the logarithm of distance to account for the nonlinear relationship between putt difficulty and success probability. Model diagnostics indicate a satisfactory fit. The generalized chi-square statistic per degree of freedom is 0.96, suggesting no material overdispersion in the data. The coefficient on log distance is negative and statistically significant (β = −1.5784), confirming the expected decline in make probability as putts become longer. The coefficient on the par indicator is small and statistically insignificant (θ = −0.0004), indicating no baseline difference between par and birdie putts at very short distances. However, the interaction between log distance and par is positive and statistically significant (δ = 0.1855), implying that par putts decline less steeply in success probability as distance increases. In other words, the par advantage widens with distance. This pattern is consistent with reference-dependent behavior under loss aversion, as players appear more resilient to increasing difficulty when attempting to avoid a loss than when pursuing a gain.

Turning to within-hole learning, the coefficient on the second-putt indicator is positive and highly significant (φ = 1.0781), indicating substantially higher baseline conversion rates for second putts relative to first putts. The interaction between log distance and second-putt status is positive but not statistically significant at conventional levels (ψ = 0.0471), suggesting that learning operates primarily as a level shift rather than increasing systematically with difficulty. Overall, the regression results confirm robust evidence of both reference-dependent performance and within-hole learning after controlling for player heterogeneity.

**Figure 11:**
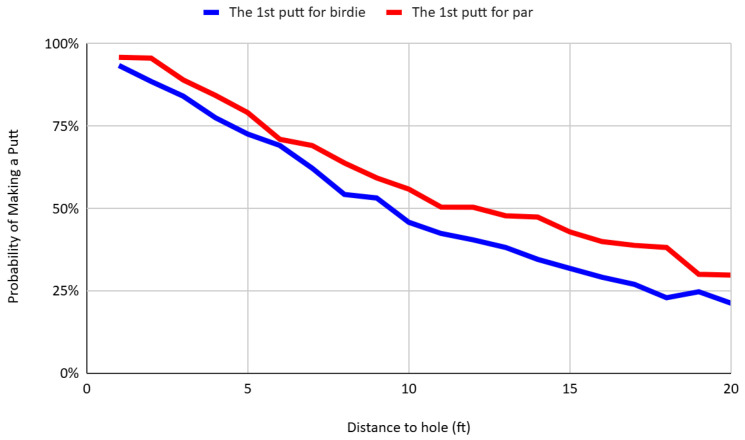
Putt success rates of first putts for birdie and par by distance.

To address the concern that estimated loss-aversion effects may partly reflect within-hole learning—for example, if par putts benefit from feedback obtained from earlier birdie attempts—the loss-aversion specification is re-examined using first putts only. Figure 11 compares conversion probabilities for first putts for birdie and par within 20 feet of the hole. Restricting the sample to first putts eliminates any influence of feedback from prior attempts on the same hole. Consistent with the full-sample evidence, par putts are converted at higher rates than equidistant birdie putts across nearly the entire distance range. The differences are statistically significant at the 1% level across most distances, with the exception of 1, 6, and 19 feet. Although the gap is smaller than in the full sample shown in Figure 3—suggesting that part of the overall difference reflects within-hole learning—the persistence of a statistically significant advantage for par putts at the first attempt demonstrates that reference-dependent performance differences arise independently of learning.

In summary, the regression evidence confirms that the visual patterns documented in Section 4 and Section 5 are statistically and economically meaningful. Using a nonlinear specification that models the logarithm of distance, the results show that par putts decline less steeply in success probability than birdie putts as distance increases, consistent with reference-dependent performance under loss aversion. The second-putt advantage remains large and precisely estimated, indicating robust within-hole learning after controlling for player heterogeneity. Moreover, restricting the analysis to first putts confirms that the observed par advantage is not driven by feedback from earlier attempts. These findings provide formal econometric support for the presence of both loss-sensitive strategic adjustment and rapid learning in professional putting.

## 5. Conclusions

This study examines the behavioral dynamics of professional golfers’ putting decisions using granular shot-level data from the KPGA Tour. By exploiting the rich structure of golf—where decisions are repeated, individually executed, and financially consequential—this paper sheds light on how loss aversion, strategic self-regulation, and adaptive learning manifest in real-world high-performance settings.

The analysis offers several key findings. First, consistent with prospect theory, golfers exhibit reference-dependent preferences, with lower conversion rates on birdie putts than par putts from equivalent distances. The regression analysis using a nonlinear specification of distance shows that par putts decline less steeply in success probability as distance increases, implying that the par advantage widens with difficulty. This asymmetry reflects the psychological salience of par as a reference point: players appear more resilient to increasing difficulty when attempting to avoid losses than when pursuing gains. The effect remains statistically significant after controlling for player fixed effects and persists when restricting the sample to first putts only, ruling out within-hole learning as the primary driver. Second, the observed variation in aggressiveness across scoring contexts highlights strategic self-regulation. Players are more aggressive on par putts, yet they modulate their risk exposure to avoid costly errors such as three-putts. The econometric evidence indicates that this differential sensitivity to distance reflects controlled adjustment under loss-framed incentives rather than purely mechanical distance management. Third, the findings reveal robust evidence of within-hole learning. Conditional on distance, second putts exhibit substantially higher conversion rates than first putts, even after controlling for player heterogeneity. Although the learning effect operates primarily as a level shift rather than increasing systematically with distance, the magnitude of the second-putt advantage is economically large and precisely estimated. Because both putts occur under nearly identical conditions, the improvement plausibly reflects genuine short-run belief updating rather than unobserved ability differences. These results are consistent with models of experience-based learning such as reinforcement learning (Erev & Roth, 1998) and experience-weighted attraction learning (Camerer & Hua Ho, 1999), where recent outcomes shape subsequent behavior.

These results advance our understanding of decision-making under uncertainty by highlighting how professional athletes integrate psychological framing, strategic planning, and adaptive learning in performance-critical environments. The golf putting context offers a natural laboratory in which individual decisions can be observed at high frequency and under meaningful incentives. More broadly, the findings underscore the value of behavioral models that account for loss sensitivity, self-regulation, and learning—not only in sports but across domains where rapid feedback and complex environments shape human behavior.

This study has several limitations. First, although the dataset provides detailed shot-level outcomes, it does not include comprehensive course-level characteristics such as green slope, surface speed, or precise pin placement. The absence of these variables limits the ability to fully disentangle psychological framing effects from the physical determinants of putting difficulty and constrains the estimation of structural models of pace choice and risk management. Future research incorporating richer course-level data could allow for more precise modeling of strategic execution and help distinguish behavioral responses from variation in environmental conditions. Second, the analysis does not explicitly account for players’ competitive standing at the time of each putt. Putting decisions made by players contending for the lead may differ systematically from those made by players positioned further down the leaderboard, as competitive incentives, risk tolerance, and psychological pressure can vary with tournament rank. As a result, heterogeneity in strategic behavior across competitive contexts is not fully captured in the current specification. Future research that integrates real-time leaderboard position or dynamic tournament incentives could shed light on how reference dependence and learning interact with competitive pressure in high-stakes environments.

## Figures and Tables

**Table 1:** List of KPGA tournaments in 2020.

Tournament	Round	Total Putts	Average Putt	Max Putt	Players
Woosung Construction Aramir CC Busan-Gyeongnam Open	4	13,212	1.72	4	156
KPGA Gunsan CC Open	4	12,650	1.65	4	156
The 63rd KPGA Championship with A-ONE CC	4	12,715	1.66	5	156
HAZZYS Golf KPGA Open with Ildong Lakes Golf Club	4	13,031	1.69	4	156
The 36th Shinhan Donghae Open	4	11,856	1.65	5	138
Hyundai Insurance KJ CHOI Invitational	4	12,103	1.74	4	132
Genesis Championship	4	10,742	1.66	4	120
BIZPLAY-Electronic Times OPEN with TAMEUS CC	3	8590	1.69	4	114
LG SIGNATURE Players Championship	4	9382	1.70	4	96

**Table 2:** Distribution of putt attempts by sequence and scoring context.

Putt Sequence	For Birdie or Eagle	For Par	For Bogey or Worse
1	41,863	17,386	2654
2	828	30,805	8140
3		117	2450
4			36
5			2

**Table 3:** Logistic Regression of Putt Success.

	Coefficient	Estimate	Standard Error	t-Stat	*p*-Value
Intercept	α	3.3824	0.0648	52.17	<0.0001
Distance	β	−1.5784	0.0228	−69.21	<0.0001
Par	θ	−0.0004	0.0860	−0.00	0.9963
Second	φ	1.0781	0.0789	13.66	<0.0001
Distance × Par	δ	0.1855	0.0334	5.55	<0.0001
Distance × Second	ψ	0.0471	0.0355	1.33	0.1842

## Data Availability

The data used in this study were obtained from CNPS, the official record-keeping agency of the Korea Professional Golfers’ Association (KPGA). The dataset was provided exclusively to the author for research purposes and is not publicly available. Access to the data is subject to CNPS approval and data-sharing policies.

## Abbreviations

The following abbreviations are used in this manuscript:

KPGA	Korea Professional Golfers’ Association
